# Non Melanoma Skin Cancer and Subsequent Cancer Risk

**DOI:** 10.1371/journal.pone.0099674

**Published:** 2014-06-17

**Authors:** Judy R. Rees, M. Scot Zens, Jiang Gui, Maria O. Celaya, Bruce L. Riddle, Margaret R. Karagas

**Affiliations:** 1 Geisel School of Medicine at Dartmouth, Department of Community and Family Medicine, Biostatistics and Epidemiology Section, Lebanon, New Hampshire, United States of America; 2 New Hampshire State Cancer Registry, Hanover, New Hampshire, United States of America; University of Pennsylvania, United States of America

## Abstract

**Introduction:**

Several studies have shown an increased risk of cancer after non melanoma skin cancers (NMSC) but the individual risk factors underlying this risk have not been elucidated, especially in relation to sun exposure and skin sensitivity to sunlight.

**Purpose:**

The aim of this study was to examine the individual risk factors associated with the development of subsequent cancers after non melanoma skin cancer.

**Methods:**

Participants in the population-based New Hampshire Skin Cancer Study provided detailed risk factor data, and subsequent cancers were identified via linkage with the state cancer registry. Deaths were identified via state and national death records. A Cox proportional hazard model was used to estimate risk of subsequent malignancies in NMSC patients versus controls and to assess the potential confounding effects of multiple risk factors on this risk.

**Results:**

Among 3584 participants, risk of a subsequent cancer (other than NMSC) was higher after basal cell carcinoma (BCC) (adjusted HR 1.40 [95% CI 1.15, 1.71]) than squamous cell carcinoma (SCC) (adjusted HR 1.18 [95% CI 0.95, 1.46]) compared to controls (adjusted for age, sex and current cigarette smoking). After SCC, risk was higher among those diagnosed before age 60 (HR 1.96 [95% CI 1.24, 3.12]). An over 3-fold risk of melanoma after SCC (HR 3.62; 95% CI 1.85, 7.11) and BCC (HR 3.28; 95% CI 1.66, 6.51) was observed, even after further adjustment for sun exposure-related factors and family history of skin cancer. In men, prostate cancer incidence was higher after BCC compared to controls (HR 1.64; 95% CI 1.10, 2.46).

**Conclusions:**

Our population-based study indicates an increased cancer risk after NMSC that cannot be fully explained by known cancer risk factors.

## Introduction

The non melanoma skin cancers (NMSC) are the most frequently diagnosed malignancies in the United States [1] with an estimated 900,000 to 1,200,000 new cases diagnosed each year. The two major types of NMSC, basal cell (BCC) and squamous cell carcinoma (SCC), have a relatively small impact on mortality but their public health impact is considerable. Dramatic increases in incidence have been documented in recent decades [2]–[4]. In New Hampshire, increases of 235% (males) and 350% (females) in SCC incidence and 80% in BCC incidence were documented among both males and females over 14 years [4].

Several studies have reported that individuals diagnosed with non melanoma skin cancers have higher subsequent or prior diagnoses of second primary malignancies by about 20–60%. [5]–[29] Record linkage studies have the advantage of a large, well-defined population base, but they generally lack detailed individual cancer risk factor data such as family history of cancer and dietary information. To our knowledge, few published reports have combined histological confirmation of NMSC, tumor registry verification of subsequent cancers, and the analysis of individual risk factor data [8], [9], [24], [30].

By virtue of their high frequency and low mortality, non melanoma skin cancers offer an excellent opportunity to study the factors that put some individuals at increased risk of multiple malignancies. We had the opportunity to elucidate the risk of multiple malignancies using a large population-based series of SCC and BCC cases and controls with individual risk factors collected through a detailed personal interview. In addition, we aimed to assess whether the excess risk of cancer after NMSC could be explained by known cancer risk factors.

## Materials and Methods

### Ethics Statement

The New Hampshire Skin Cancer Study (NHSCS) and the additional work described here were approved by the Committee for the Protection of Human Subjects of Dartmouth College. Participants in the NHSCS underwent a written, informed consent process at enrollment. Use of data from the New Hampshire State Cancer Registry was approved by the New Hampshire Department of Public Health Services. Confidentiality was also protected by use of a de-identified dataset in the statistical analyses.

### New Hampshire Skin Cancer Study

The New Hampshire Skin Cancer Study (NHSCS) was first conducted as a population-based case-control study of non melanoma skin cancers (NMSC) diagnosed between July 1, 1993 and June 30, 2002 (New Hampshire residents), obtained through intensive surveillance at dermatologists’ offices and pathology laboratories serving the state. Case selection criteria earlier in the study tended to oversample patients with SCC relative to their incidence in the population, and in later years there was oversampling of cases diagnosed before the age of 51 years (“early-onset”), or with multiple concomitant BCCs. Cases with both incident BCC or SCC during the enrollment phase were eligible based on either diagnosis, as dictated by the sampling methods of the parent study. Controls were selected from NH residents provided by the NH Department of Transportation, frequency matched on age and sex. [31] Although the skin cancer study includes individuals with all ethnic backgrounds, they were recruited from the New Hampshire population which was ∼98% white (1990 US Census). The date of diagnosis, or a matched comparable date generated for controls, served as the reference date for this study. Collection of the data occurred in several phases, with the addition of new variables as the study progressed. Detailed risk factor information was obtained from personal interviews on 2,713 individuals with NMSC (SCC 1,170; BCC 1,543), and 1,416 age- and sex-matched controls. This included information about smoking, education, skin type, lifetime sun exposure habits including number of painful sunburns, skin type, number of nevi, body mass index, weight gain since age 18, smoking, alcohol consumption, nutritional information including dietary and supplementary intake of vitamin D, folic acid, and multivitamins, history of cancer, radiotherapy, prescription and over the counter drug use, and family history of cancer including age at diagnosis and cancer site. Toenail arsenic was also measured and analyzed using instrumental neutron activation analysis. For the purposes of the analyses described here, the original groups (BCC, SCC and controls) were followed as a retrospective cohort.

### New Hampshire State Cancer Registry

The New Hampshire State Cancer Registry (NHSCR) is a population-based database of incident, reportable cancers containing, on average, ∼6,500 verified new cases annually among the New Hampshire population of 1.2 million. Incidence data for 1995 onwards meet the standards of the North American Association of Central Cancer Registries for quality and completeness [32]. Case ascertainment from 1986 through 1995 is partially complete, and some information on non-reportable cases (diagnosed before 1986 or before the patient took up residence in New Hampshire) is held by NHSCR in separate databases. The New Hampshire State Cancer Registry annually links its database with the National Death Index and with death certificate files from the state of New Hampshire, to identify cancer-related deaths and their dates. The NHSCR database is checked annually for data quality and consistency using nationally accepted automated validation tools, and the database from 2000 onwards is estimated to be over 99% complete, according to formulae applied by the National Program for Cancer Registries (NPCR). These NPCR evaluations began in 1995; between 1995 and 1999, NHSCR completeness estimates ranged between 88% and 95%.

### Exclusions of Individuals with a Prior History of Cancer

We excluded from the main analyses any individual with a prior history of cancer other than skin cancer (NMSC and melanoma). This exclusion encompassed any non-skin cancer diagnosed before the reference date identified either through NHSCR records or via self-report at the study enrollment interview. Self-reports of major cancers have previously been shown to be fairly reliable [33], [34] and allowed us to identify cancers that would not have been ascertained by our registry (i.e., occurred outside New Hampshire or before 1995). For consistency between self-reported and NHSCR cancers, registry-reported *in situ* malignancies (excluding cervix and prostate) were included among “prior” cancers.

### Ascertainment of Cancer Following NMSC

To identify cancers that were diagnosed after the reference date, we linked the NHSCS database with the NHSCR incident cancers database for diagnosis through 2009, which, on July 23^rd^, 2010, contained 149,523 individuals. We used LinkPlus software [35] to conduct probabilistic matching based on social security number, last name, first name, date of birth and sex. The software listed the linked records in order of the estimated probability of a true match and one author (JR) manually reviewed linkage variables alongside street address, zip code and telephone number as additional means of linkage verification. Because the definitions of reportable cancers changed over time, we based our definition of subsequent cancers on those reportable in 2008 [36]. Subsequent cancers were defined as cancers reported to the NHSCR with stage 1 or higher (invasive disease), or any bladder cancers (including in situ, stage 0); data on malignant melanomas were collected, but melanomas were examined separately in some analyses.

### Identification of Deaths

We linked both the NHSCR and NMSC databases with the New Hampshire death certificate database to identify deaths from 1993 through 2009. We again used LinkPlus combined with manual review to determine deaths among study participants. In addition, we linked with the National Death Index (NDI) through 2009 to identify deaths nationwide. Death data were used to provide censor dates in the survival analysis, and to identify potential cancers that were not identified from cancer registry data.

### Statistical Analysis

Although the parent study was a case control design, the data were analyzed for this study in a cohort design, using follow-up information from the date of enrollment for participants with BCC or SCC, and controls. The endpoint of our analysis was the time from the reference date to diagnosis of first cancer. Participants who did not develop cancer were censored on the date of death or at the end of follow-up (December 31, 2009), whichever came first. We used Cox models to determine the hazard ratios (HRs) and 95% confidence intervals (CI) associated with development of a subsequent cancer for BCC and SCC cases (separately) versus controls. We also performed stratified analyses by sex and by age group (<60 or ≥60 years) and restricted to invasive cancers. For all analyses, the HRs were adjusted for known the cancer risk factors, age, sex, and smoking status (never, former, current). We plotted the survival curves in the BCC, SCC and control groups to check the proportional hazards assumption and separately assessed the hazard functions. We examined the contribution of additional variables to the model (e.g., sun exposure, body mass index) by assessing the change in the primary measure of effect; thus, we included variables in the age-, sex-, smoking-adjusted model which led to a 10% or greater change in the hazard ratio for BCC, SCC or NMSC (BCC and SCC combined). [37] We further developed models for site-specific analyses, in which we redefined the end point as diagnosis of the specific cancer (e.g. time to first melanoma, time to first prostate cancer) whether or not there were intervening cancers of a different kind. We conducted sensitivity analyses (i) after excluding subsequent cancers diagnosed within the first year after the referent NMSC, (ii) after excluding individuals with a prior melanoma; (iii) defining prior cancers only by self report; and (iv) defining prior cancers only via the registry. We also separately assessed the risk of subsequent cancer in those who had been excluded from the primary analysis because of a prior cancer (File S1).

## Results

The skin cancer study provided interview data from a total of 4,223 individuals followed for a mean of 10.9 years (range 0.8 to 17.0). From the 1,600 individuals enrolled as a result of a diagnosis with BCC, 1,125 with SCC and 1,498 controls (Table 1), we excluded 642 participants with prior internal cancers based on self-report or registry confirmation from the analysis. These exclusions represented 10.5% of those without a history of NMSC, 14.8% of those with a history of BCC and 22.0% with SCC.

**Table 1:** Follow-up of participants and their subsequent cancers after non melanoma skin cancer.

		Controls			BCC^1^			SCC^1^		
		Men	Women	All	Men	Women	All	Men	Women	All
Participants in the study		833	665	1,498	813	787	1,600	700	425	1,125
	Excluded due to prior non skin cancer^2^	73 (8.8%)	84 (12.6%)	157 (10.5%)	130 (16.0%)	107 (13.6%)	237 (14.8%)	160 (22.9%)	88 (20.7%)	248 (22.0%)
	Included in the analyses	760 (91.2%)	581 (87.4%)	1,341 (89.5%)	683 (84.0%)	680 (86.4%)	1,363 (85.2%)	540 (77.1%)	337 (79.3%)	877 (78.0%)
	Mean years of follow-up per person	10.9	11.1	11.0	11.3	11.1	11.2	10.1	10.7	10.4
	Participants with cancer diagnosed afterreference date	121	65	186	140	73	213	110	51	161
Tumors diagnosed afterreference date^3^		134	67	201	175	84	259	130	56	186
Stage of tumors^3^	In situ (stage 0 except bladder)	7 (5.2%)	4 (6.0%)	11 (5.5%)	15 (8.6%)	18 (21.4%)	33 (12.7%)	13 (10.0%)	9 (16.1%)	22 (11.8%)
	Invasive (stage 1–4, stage 0 bladder)	100 (74.6%)	58 (86.6%)	158 (78.6%)	136 (77.7%)	55 (65.5%)	191 (73.7%)	96 (73.8%)	40 (71.4%)	136 (73.1%)
	Unstaged	8 (6.0%)	2 (3.0%)	10 (5.0%)	13 (7.4%)	6 (7.1%)	19 (7.3%)	9 (6.9%)	3 (5.4%)	12 (6.5%)
	Unknown	19 (14.2%)	3 (4.5%)	22 (10.9%)	11 (6.3%)	5 (6.0%)	16 (6.2%)	12 (9.2%)	4 (7.1%)	16 (8.6%)

1 Of 2,725 cases, 82 (3%) had both a BCC and SCC diagnosed within 30 days of the reference date. Of these, oversampling for SCC cases led 79 to an SCC classification.

2 Participants with cancer of any organ except the skin, diagnosed before the reference date, were excluded from the main analyses. Relative to controls, the odds ratio for a history of non-skin cancer in those with BCC was, adjusted for age, sex and smoking: odds ratio 1.74 (95% CI 1.40, 2.18) and for SCC, OR 1.99 (95% CI 1.59, 2.89).

3 Total exceeds number of participants with subsequent cancer because a participant may be diagnosed with multiple primary tumors.

The mean age of the remaining 3,584 participants included in the analysis was 57.5 (standard deviation 11.7), and 55% were male. During follow-up, 562/3584 (15.7%) eligible participants died from any cause; 203/1,341 (15.1%) of controls, 170/1,363 (12.5%) of those with BCC and 189/880 (21.6%) with SCC. A total of 560 individuals (13.7%) developed 646 subsequent cancers during follow-up; 485 developed 1 type of cancer; 65 developed 2 types of cancer; 10 developed 3 or more types of cancer.

After adjusting for age, sex and smoking (never, former, current), the risk of a subsequent cancer (excluding NMSC) was higher following a BCC diagnosis compared to controls (HR 1.40 [95% CI 1.15, 1.71]). The hazard ratios for BCC were significantly elevated among men (HR 1.55 [95% CI 1.21, 1.99]) but not women (HR 1.14 [0.81, 1.60]) and among participants aged 60 or more at enrolment (HR 1.43 [95% CI 1.14, 1.80]) but not among those younger than 60 years (HR 1.31 [95% CI 0.88, 1.95]). Following SCC, we observed a more modest, not statistically significant increase overall (HR 1.18 [95% CI 0.95, 1.46]). However, the risk of subsequent cancer after SCC was substantially increased when diagnosed before age 60 (HR 1.96 [95% CI 1.24, 3.12]), whereas no effect was seen among those diagnosed at age 60 or older (HR 1.00 [95% CI 0.79, 1.28]) and this difference was statistically significant (p<0.003). In contrast, significant interactions were not seen between age group and BCC, nor between sex and either BCC or SCC (Table 2). The proportional hazards assumption was supported by review of survival plots and hazard functions for the three groups (BCC, SCC and control).

**Table 2:** Hazard ratios (95% confidence intervals) for subsequent cancers (all, and invasive cancers only) following basal cell and squamous cell skin cancers compared to controls.

	BCC				SCC			
	N = 1,363				N = 880			
	All cancers^1^		Invasive cancers^2^		All cancers^1^		Invasive cancers^2^	
	Number withcancer	HR (95% CI)	Number withinvasive cancer	HR (95% CI)	Number withcancer	HR (95% CI)	Number withinvasive cancer	HR (95% CI)
All participants	213	**1.40 (1.15, 1.71)**	182	**1.37 (1.11, 1.70)**	161	1.18 (0.95, 1.46)	131	1.09 (0.86, 1.38)
Men	140	**1.55 (1.21, 1.99)** 3	123	**1.60 (1.23, 2.09)**	110	1.17 (0.90, 1.52)^4^	91	1.12 (0.84, 1.49)
Women	73	1.14 (0.81, 1.60)^3^	59	1.02 (0.70, 1.47)	51	1.22 (0.84, 1.79)^4^	40	1.06 (0.70, 1.61)
<60 years old	64	1.31 (0.88, 1.95)^3^	51	1.16 (0.75, 1.78)	39	**1.96 (1.24, 3.12)** 4	30	1.64 (0.98, 2.72)
≥60 years old	149	**1.43 (1.14, 1.80)** 3	131	**1.45 (1.13, 1.85)**	122	1.00 (0.79, 1.28)^4^	101	0.96 (0.74, 1.25)

Hazard ratios are based on time to subsequent cancer compared to controls, adjusted for age at reference date, sex and smoking; stratified models are similarly adjusted except for the stratification variable. Where shown in bold, HR is statistically significant (alpha = 0.05).

1 All cancers: invasive plus in situ cancers of sites other than cervix and prostate.

2 Invasive cancers: stage 1–4 cancers and stage 0 bladder.

3 The HR associated with BCC did not vary significantly by sex or by age group.

4 The HR associated with SCC did not vary significantly by sex, but was significantly higher in those aged <60 than those aged ≥60 (p<0.003).

Factors other than a history of NMSC that were related to risk of subsequent cancer included increasing age (HR 1.06; 95% CI 1.05, 1.07), male sex (HR 1.44; 95% 1.21, 1.73) and current cigarette smoking (HR 1.53; 95% 1.21, 1.94, Table S1 in File S1). Occupational sun exposure was associated with a statistically significantly lower risk of cancer after BCC (e.g. highest vs. lowest quartile HR 0.72 (95% CI 0.53, 0.97), but was not retained in the model as it did not impact the association between BCC and subsequent cancer. Other factors that appeared to be unrelated to risk of subsequent cancer included education, BMI, weight gain since age 18, skin reaction to chronic sun exposure, lifetime warm month sun exposure, family history of cancer, folate intake, and toenail arsenic concentration and skin reaction to acute sun exposure, lifetime painful sunburns, self reported number of nevi on the back, BMI at age 18, vitamin D intake; warm month cumulative sun exposures as adult and as child; proportion of sun exposure that was recreational; history of radiotherapy; regular use of oral steroids, aspirin, acetaminophen, non-steroidal anti-inflammatory agents; age first started smoking; coffee, tea or alcohol consumption; family history of cancers of all major sites (each of which was tested separately if female, male, age <50, age >50) (data not shown). We further examined risks of subsequent melanoma and other specific types of cancers following NMSCs (Table 3). Risk of both melanoma and prostate cancers were higher after NMSC compared with controls. In an analysis of time to diagnosis of melanoma, we found a 3-fold increase in risk after BCC (HR 3.28; 95% CI 1.66, 6.51), after adjustment for age, sex and smoking, skin reaction to chronic sun exposure and family history of non melanoma skin cancer. The hazard ratio for SCC versus controls (HR 3.62; 95% CI 1.85, 7.11) adjusted for age, sex, smoking, skin reaction to chronic sun exposure was likewise elevated. Family history of NMSC was unrelated to cancer risk following an SCC and therefore not included as a covariate this model. For all NMSC combined, a family history of NMSC was associated with an increased risk of subsequent melanoma (HR 1.60; 95% CI 1.01, 2.52). Following BCC, a lower risk of subsequent melanoma was seen in former smokers when compared to never smokers (HR 0.49; 95% CI 0.26, 0.91; Table S1 in File S1). A similar pattern was seen after SCC, but without statistical significance. Excluding melanomas diagnosed within 12 months of the referent NMSC did not diminish the hazard associated with NMSC status (data not shown). The hazard ratios for melanoma after BCC were higher among older patients ≥60 years (HR 5.24; 95% CI 1.96, 14.01) than younger ones (HR 1.76; 95% CI 0.67, 4.61). A similar pattern was seen in SCC (Table 3). After excluding melanoma, the hazard ratios reflecting cancer risk after BCC and SCC were lower, but still statistically significantly increased among men after BCC and among those aged <60 at the time of diagnosis of SCC (Table 3).

**Table 3:** Site-specific cancers risk after basal cell and squamous cell skin cancers versus controls.

Cancer site		Controls	BCC vs controls		SCC vs controls	
		N = 1341	N = 1,363		N = 880	
		Number withcancer	Number withcancer	HR (95% CI)	Number withcancer	HR (95% CI)
All cancers	All	186	213	**1.40 (1.15, 1.71)**	161	1.18 (0.95, 1.46)
	Men only	121	140	**1.55 (1.21, 1.99)**	110	1.17 (0.90,1.52)
	Women only	65	73	**1.14 (0.81, 1.60)**	51	1.22 (0.84, 1.79)
	<60 y	40	64	**1.31 (0.88, 1.95)**	39	**1.96 (1.24, 3.12)**
	≥60 y	146	149	**1.43 (1.14, 1.80)**	122	1.00 (0.79, 1.28)
All cancers exceptmelanoma	All	177	180	**1.24 (1.01, 1.54)**	136	1.01 (0.81, 1.27)
	Men only	115	117	**1.37 (1.05, 1.78)**	91	0.98 (0.74, 1.30)
	Women only	62	63	1.03 (0.72, 1.48)	45	1.11 (0.74, 1.30)
	<60 y	35	50	1.17 (0.76, 1.82)	29	1.67 (0.99, 2.81)
	≥60 y	142	130	1.27 (1.00, 1.61)	107	0.89 (0.69, 1.15)
Melanoma	All	15	63	**3.28 (1.66, 6.51)** 1	42	**3.62 (1.85. 7.11)** 2
	Men only	12	46	**3.19 (1.48, 6.91)**	32	**3.53 (1.63, 7.62)**
	Women only	3	17	3.87 (0.82, 18.22)	10	**4.29 (1.05, 17.53)**
	<60 y	7	30	1.76 (0.67, 4.61)	11	2.15 (0.76, 6.10)
	≥60 y	8	33	**5.24 (1.96, 14.01)**	31	**4.32 (1.75, 10.67)**
Prostate	All	42	56	**1.64 (1.10, 2.46)**	32	0.93 (0.59, 1.49)
	<60 y	5	15	2.49 (0.90, 6.88)	5	1.58 (0.44, 5.59)
	≥60 y	37	41	1.51 (0.97, 2.36)	27	0.84 (0.51, 1.39)
Lung		33	27	1.14 (0.68, 1.90)	16	0.69 (0.38, 1.25)
Female breast		20	24	1.13 (0.62, 2.06)	16	1.24 (0.63, 2.45)
Colorectal		24	19	0.92 (0.50, 1.69)	17	0.82 (0.43, 1.56)
Bladder		11	13	1.47 (0.65, 3.31)	9	1.02 (0.42, 2.48)
Non Hodgkin lymphoma		7	6	1.08 (0.36, 3.26)	9	1.59 (0.59, 4.31)
Uterus		11	5	0.42 (0.15, 1.23)	4	0.63 (0.19, 2.07)

Hazard ratios refer to NMSC status (vs controls), adjusted for age at reference date, sex and smoking.

1 The melanoma models for BCC are adjusted for age at reference date, sex, smoking, skin reaction to chronic sun exposure and family history of NMSC.

2 The melanoma models for SCC are adjusted for age at reference date, sex, smoking, skin reaction to chronic sun exposure.

Stratified models are similarly adjusted except for the stratification variable.

An increased risk of prostate cancer was observed after BCC (HR 1.64; 95% CI 1.10, 2.46) but not SCC adjusted for age, sex and smoking. These hazard ratios were not appreciably altered by other risk factors, nor by exclusion of prostate cancers diagnosed within 12 months of the referent lesion (data not shown). We examined risk of subsequent cancers in several subgroups. For individuals with multiple BCC (defined as two or more tumors within 30 days), the adjusted HR for all subsequent cancers (including *in situ*) relative to all controls was 1.59 (95% CI 1.11, 2.27), and 1.39 (0.93, 2.08) for only invasive subsequent cancers. When an individual’s referent BCC occurred before age 50, the adjusted HR was similar to that seen in the primary analysis (HR 1.40; 95% CI 0.79, 2.49) for all subsequent cancers but lower (HR 1.09; 95% CI 0.59, 2.01) for invasive subsequent cancers.

Overall, approximately 1% of NMSC patients were found to have another malignancy within a year of their diagnosis. During the first year of observation, an internal cancer or melanoma was diagnosed in 12/880 (1.4%) SCC patients, 9/1363 (0.7%) BCC patients, and only 1/1341 (<0.1%) controls (data not shown).

In sensitivity analyses, we obtained similar results after: (i) excluding cancers diagnosed within the first year, (ii) excluding individuals from the analysis who had a prior melanoma (as well as other non skin cancer); (iii) defining prior cancers by self report only or (iv) by registry report only (File S1).

## Discussion

In our study, we identified a significantly increased cancer risk after BCC that could not be explained by a variety of environmental, nutritional or behavioral risk factors or by family history of cancer. The increased cancer risk after SCC appeared to be largely confined to those with SCC diagnosed before the age of 60. In our population, the strongest association was for subsequent melanoma of the skin, supporting common susceptibility and exposures such as ultraviolet light in the etiology of skin malignancies. However, after excluding melanoma, there remained a statistically significant increased risk of subsequent malignancy after BCC, especially among men. Any attempt to explain the increased cancer risks after NMSC must consider the environmental, genetic, and personal characteristics that could predispose to both NMSC and other cancers. By adding important explanatory factors to the models, we would expect to see a reduction in the adjusted hazard ratios for risk of cancer after NMSC, but NMSC-related hazard remained in our study, despite the inclusion of many potential risk factors. This suggests the need for studies of more detailed risk factor data including genetic analyses in large populations. [38], [39].

A few other published studies have incorporated histological confirmation of NMSC, cancer registry confirmation of subsequent cancer, and multivariable adjustment for individual risk factors. The largest of these was based on 36,102 individuals with NMSC from US prospective cohorts followed by Song et al through postal questionnaires that collected individual level risk factors, although estimates of cumulative sun exposure or family history of NMSC were not available. [24] In multivariable analyses, they found that SCC was associated with a 24% increase in risk in women but little to no association in men; BCC was associated with a 25% increase in risk in women and 17% in men. The increased risk after NMSC in that study was seen among never and former smokers but not among current smokers, which raises questions about heterogeneity in the mechanism of carcinogenesis. That is, an individual with SCC that arose primarily due to smoking may have a different causal pathway to subsequent cancer than an individual with SCC that arose through other mechanisms, such as an intrinsic susceptibility to cancer. Song et al also detected no substantial differences in the association between NMSC and subsequent cancer by ultraviolet light (UVL) exposure at their place of residence, BMI or age. In contrast, we found associations between NMSC and subsequent cancer in those aged <60 (but not in those ≥60) at diagnosis of SCC, and among those aged ≥60 (but not in those <60) at diagnosis of BCC. Others have also found higher risks of subsequent cancer after NMSC in younger age groups. [13], [21] Studies of Kaiser Permanente patients reported estimates of relative risk closer to ours, based on 822 patients with in situ or invasive SCC (HR 1.4; 95% CI 1.2, 1.6) [8], and on 3164 patients with BCC (HR 1.2; 95% CI 1.1, 1.4) [9]. Chen studied 165 individuals with SCC, 513 with BCC, 60 with both and 31 with unknown subtype from the volunteer-based CLUE II cohort. A higher relative risk of cancer associated with prior NMSC (HR 1.99; 95% CI 1.70, 2.33) was observed than in our population-based study [30]. None of these studies, including our own, could explain away all of the NMSC-related risk in terms of individual risk factor data.

Other than NMSC, cancers related to UVL exposure, or suspected to be so, include malignant melanoma, cancer of the lip, non-Hodgkin’s lymphoma and leukemias; these are among the cancers that appear to be more common after NMSC in prior studies [11], [15], [18]–[20], [25]–[27], [40], [41]. When assessing all subsequent cancers together, we did not find any significant changes in risk associated with skin type, number of lifetime painful sunburns, or the number of hours spent outdoors during warm months, nor any modification of the increased risk associated with NMSC. The amount of time spent outdoors in an occupational setting was associated with a lower risk of subsequent cancer, after adjustment for age, sex and smoking, but, given the lack predictive value of other sun exposure measures, it seems likely that this may represent confounding by other lifestyle factors. The increased risks observed also persisted after accounting for family history of cancer, including having a first degree relative diagnosed with cancer before age 50. Although arsenic in well water is a known concern in New Hampshire, we did not find an association between toenail arsenic concentration and overall cancer risk, nor any impact in our models. [42].

While our study did not indicate that sun exposure affected non skin cancer risk, it is possible that the sample size may have been too small to do so, especially for specific types of cancers. However, we did detect an excess risk of melanoma following both BCC and SCC with significantly increased melanoma risk in participants reporting a family history of NMSC, but not a family history of melanoma which is a known risk factor for melanoma. [43] Melanoma risk was also increased among individuals who peel or develop moderate tans upon chronic sun exposure (compared to those who develop a deep tan). Former smokers had a significantly lower risk of melanoma after NMSC and a risk reduction was seen in current smokers but this could have been due to chance; several other studies have reported reduced melanoma risk in smokers, but no adequate explanation for this has been put forward except for the possibility of bias caused by competing risk due to unknown confounders. [44] Family history of melanoma did not contribute significantly to the model as in other studies, [45] but the numbers with such a history in our cohort were much smaller. We found that the excess risk of melanoma after BCC or SCC remained after accounting for known risk factors such as sun sensitive skin type. On balance, our findings raise the possibility of unmeasured or unknown shared genetic risk factors for melanoma and BCC.

In the site-specific analysis, we further found that BCC but not SCC was associated with an increased risk of subsequent prostate cancer. Again, we did not identify any additional risk factors that affect our risk estimates, or could explain this association. However, our approach was potentially limited by lack of statistical power and our method of including site-specific cancers even if intervening cancers occurred. For example, if a BCC were followed by a breast cancer and then a lung cancer, the lung cancer would be included in the site-specific analysis despite the possibility that treatment of the breast cancer may have played a role in the lung cancer etiology.

Participants with NMSC in this study were selected from a population-based surveillance program, and are therefore expected to represent reasonably well the general population of individuals diagnosed with NMSC, although it should be noted that the relative proportions of SCC and BCC patients in our study do not reflect population incidence due to oversampling of SCC patients in the parent study. In particular, 3% of cases were diagnosed with both a BCC and SCC within a 30-day period, and these were over-represented in the SCC group. We could not assess the proportions of patients who were diagnosed with both cancers during the course of the study or during their lifetime, although it would have been interesting to analyze this group’s subsequent risks separately. An additional limitation is that controls may have subsequently developed NMSC during a decade of observation, and would therefore be misclassified as controls; this would tend to bias the results of our study towards the null, and reduce our ability to identify true associations between NMSC and the risk of subsequent cancer. Although the expected frequency of subsequent NMSC among controls is relatively high because NMSC are common cancers, fewer than 0.1% of controls developed a major cancer during the first year after the reference date, a figure comparable to the similarly aged US population [46]. A higher proportion of those with NMSC – almost 1% - developed another type of cancer during that first year. While it seems likely that increased medical surveillance after NMSC might account for this observation, we did not find clear evidence that earlier cancers were of an earlier stage, and exclusion of cancers diagnosed within one year did not materially change our results. Irrespective of whether these findings reflect detection bias, it may be useful for dermatologists to know that 1.4% of our patients diagnosed with SCC, and 0.7% of those with BCC had an internal cancer or melanoma that could be diagnosed within 12 months after an NMSC diagnosis.

Another limitation is that we were unable to assess the impact of race because the study population based in New Hampshire lacks racial diversity. Previously, a large cross-sectional study found that self-reported NMSC was more likely to be associated with other self-reported cancers in black than white women [23]. However, it is unclear how much of this apparent effect modification by race reflects a stronger association between NMSC and subsequent cancer in black women or simply differences in patterns of self-reporting of cancers [23]. The ascertainment of cancers before 1995 is a limitation of our study, because cancer registry data before 1995 were less complete. In addition, cancer case ascertainment by the NH State Cancer Registry depended on continued residence in New Hampshire or another state (including its immediate neighbors) that reports to New Hampshire. Sensitivity analyses using different definitions of prior cancer, including self-report, confirmed our major findings (data not shown). Finally, our results include the results of many comparisons; as we did not statistically address the impact of multiple testing, it is possible that some of our findings may be the result of chance.

In summary, individuals with NMSC and no prior history of non-skin cancer were more likely to develop another cancer following a NMSC diagnosis, particularly melanomas. Detailed individual risk factors other than age, sex and smoking could not explain this increase in cancer risk overall. Sun sensitive skin type and family history of NMSC explained a small fraction of the excess melanoma risk after NMSC, but adjustment for several other known and putative cancer risk factors did not remove the association between NMSC and subsequent melanoma or other cancers. Understanding the shared risk factors that contribute to multiple malignancies may lead to new etiologic insights. In particular, larger studies incorporating detailed genetic data may help to identify NMSC patients at greatest risk for subsequent cancers and who may benefit from more intensive screening.

## Supporting Information

File S1 **Supplementary data.**
(DOCX)
