# Supplementary material for: Non Melanoma Skin Cancer and Subsequent Cancer Risk
Source: PLoS One. 2014 Jun 17;9(6):e99674. doi: 10.1371/journal.pone.0099674 (PMC4061037; doi:10.1371/journal.pone.0099674)
Supplement: File S1 — Supplementary data. (DOCX) [file pone.0099674.s001.docx]

**Supplementary data**

**1. Risk of subsequent cancer in the New Hampshire Skin Cancer Study**

As described in Methods, models were developed to assess the impact of various personal characteristics on the risk of subsequent cancer after non melanoma skin cancer. Selected variables are shown in Table S1 and the full list of variables tested is given in the text.

**2. Sensitivity analyses**

We conducted the following sensitivity analyses: (i) Excluding 25 cancers diagnosed within the first year yielded similar results to the primary analyses (BCC HR 1.35; 95% CI 1.10, 1.65; SCC HR 1.10; 95% CI 0.88, 1.37). (ii) Excluding 154 individuals from the analysis who had a prior melanoma (as well as other non skin cancer) made little difference to the primary results (BCC HR 1.35; 95% CI 0.86, 2.11; SCC HR 1.17; 95% CI 0.94, 1.46). (iii) Defining prior cancers by self report (N=589) again left our primary results essentially unchanged (BCC HR 1.40; 95% CI 1.15, 1.71; SCC HR 1.20; 95% 0.97, 1.49). (iv) Defining prior cancers as those identified only via the New Hampshire State Cancer Registry (N=273), the risk associated with SCC was somewhat higher (HR 1.27; 05% CI 1.04, 1.55), but the risk after BCC was largely unchanged (HR 1.39; 95% CI 1.15, 1.67).

When we separately assessed those who had been excluded from the primary analysis because of a prior non-skin cancer, there was an increased risk of subsequent cancer associated with SCC (N=243) (HR 1.74; 95% CI 1.13, 2.68) not seen among those without a prior cancer (HR 1.18; 95% CI 0.95, 1.46). After BCC, subsequent risk of cancer was similar in those with a prior cancer (N=234) (HR 1.35; 95% CI 0.86, 2.11) to that seen among those without prior cancer (HR 1.59; 95% CI 1.11-2.27).

**3. Subsequent melanoma after NMSC.**

Table S2 shows the models describing the melanoma risk of melanoma after BCC or SCC included age, sex, smoking and skin reaction to chronic sun exposure; the model for BCC also included family history of NMSC.

Table S1. Risk of subsequent cancer associated with selected characteristics in the New Hampshire Skin Cancer Study

|  | | Controls | BCC and controls | | SCC and controls | |
| --- | --- | --- | --- | --- | --- | --- |
|  |  | N = 1,341 | N = 1,363 | HR^2^ (95% CI) | N = 880 | HR^2^ (95% CI) |
| Age (years) | Mean (SD) | 57.4 (11.9) | 54.2 (11.9) | 1.40 (1.15, 1.71) | 62.9 (8.9) | 1.18 (0.95, 1.46) |
| Sex | Male | 760 | 683 | 1.00 | 540 | 1.00 |
|  | Female | 581 | 680 | **0.69 (0.56, 0.86)** | 340 | **0.75 (0.60, 0.94)** |
| Smoking | Never | 501 | 614 | 1.00 | 306 | 1.00 |
|  | Former | 240 | 205 | 0.98 (0.78, 1.23) | 134 | 0.99 (0.77, 1.26) |
|  | Current | 594 | 536 | **1.57 (1.19, 2.08)** | 435 | **1.67 (1.24, 2.25)** |
|  | Unknown | 6 | 8 |  | 5 |  |
| Other putative cancer risk factors not retained in model: |  |  |  |  |  |  |
| Highest education | High school | 565 | 410 | 1.00 | 347 | 1.00 |
|  | Any college | 494 | 558 | 1.06 (0.84, 1.32) | 300 | 0.90 (0.72, 1.15) |
|  | Post graduate | 272 | 483 | 0.80 (0.60, 1.05) | 223 | 0.79 (0.59, 1.06) |
|  | Unknown | 10 | 12 |  | 10 |  |
| Baseline body mass index | 18.5 - <25 | 288 | 362 |  | 216 | 1.00 |
|  | <18.5 | 6 | 15 | 0.58 (0.08, 4.21) | 8 | 0.70 (0.10, 5.12) |
|  | 25 - <30 | 307 | 265 | 1.21 (0.86, 1.71) | 268 | 1.10 (0.79, 1.54) |
|  | ≥30 | 166 | 131 | 0.84 (0.53, 1.33) | 107 | 1.04 (0.68, 1.59) |
|  | Unknown | 574 | 590 |  | 281 |  |
| Weight gain (kg) since 18y | -36.3 - 5.9 | 179 | 217 | 1.00 | 145 | 1.00 |
|  | 5.9 - 12.2 | 191 | 203 | 1.45 (0.92, 2.29) | 151 | 1.20 (0.77, 1.87) |
|  | 12.2 - 20.4 | 191 | 211 | 1.41 (0.90, 2.21) | 168 | 1.36 (0.89, 2.06) |
|  | 20.4 - 74.8 | 205 | 141 | 1.13 (0.70, 1.81) | 134 | 1.14 (0.73, 1.76) |
|  | Unknown | 575 | 591 |  | 282 |  |
| Skin reaction to chronic sun exposure | Deep tan | 396 | 234 | 1.00 | 143 | 1.00 |
|  | Moderate tan | 620 | 613 | 1.16 (0.90, 1.50) | 415 | 1.15 (0.88, 1.52) |
|  | Peel | 255 | 386 | 1.17 (0.87, 1.58) | 215 | 0.89 (0.64, 1.26) |
|  | No tan | 56 | 116 | 0.93 (0.57, 1.50) | 94 | 1.10 (0.71, 1.73) |
|  | Unknown | 14 | 14 |  | 13 |  |
| Lifetime warm month sun (1,000 hrs) | <10.5 | 314 | 360 | 1.00 | 126 | 1.00 |
|  | 10.5 - 15.0 | 280 | 349 | 1.12 (0.81, 1.54) | 171 | 0.84 (0.57, 1.23) |
|  | 15.0 - 20.9 | 302 | 280 | 1.00 (0.72, 1.38) | 217 | 0.84 (0.58, 1.22) |
|  | 20.9 - 48.1 | 286 | 245 | 0.98 (0.70, 1.36) | 269 | 0.91 (0.64, 1.31) |
|  | Unknown | 159 | 129 |  | 97 |  |
| Occupational warm month sun (1,000 hrs) | <1.6 | 274 | 367 | 1.00 | 174 |  |
|  | 1.6 - 3.9 | 308 | 342 | **0.68 (0.50, 0.93)** | 165 | 0.77 (0.54, 1.11) |
|  | 3.9 - 8.8 | 311 | 300 | 0.77 (0.57, 1.03) | 204 | 0.81 (0.58, 1.13) |
|  | 8.8 - 41.5 | 314 | 250 | **0.72 (0.53, 0.97)** | 251 | 0.80 (0.57, 1.12) |
|  | Unknown | 124 | 104 |  | 86 |  |
| Family history cancer^2^ | No | 670 | 688 | 1.00 | 412 | 1.00 |
|  | Yes <50 yr | 183 | 168 | 1.09 (0.82, 1.47) | 123 | 1.01 (0.74, 1.38) |
|  | Yes ≥50 yr | 411 | 451 | 0.98 (0.78, 1.23) | 310 | 1.01 (0.79, 1.28) |
|  | Yes, unknown | 15 | 15 |  | 9 |  |
|  | Unknown | 62 | 41 |  | 30 |  |
| Daily folate intake (mcg) | 65-462 | 129 | 143 | 1.00 | 85 | 1.00 |
|  | 462-878 | 143 | 131 | 0.69 (0.38, 1.24) | 77 | 1.27 (0.70, 2.30) |
|  | 878-1308.5 | 131 | 129 | 0.69 (0.38, 1.24) | 93 | 1.00 (0.53, 1.86) |
|  | 1308.5-7333 | 127 | 133 | 0.94 (0.55, 1.62) | 94 | 1.48 (0.83, 2.63) |
|  | Unknown | 811 | 827 |  | 531 |  |
| Log toenail arsenic (ppb) | -8 – 0.06 | 306 | 291 | 1.00 | 238 | 1.00 |
|  | 0.06 – 0.09 | 316 | 303 | 0.98 (0.74, 1.29) | 223 | 1.09 (0.81, 1.47) |
|  | 0.09 – 0.13 | 304 | 335 | 1.08 (0.82, 1.43) | 199 | 1.16 (0.85, 1.58) |
|  | 0.13–3.26 | 308 | 352 | 0.90 (0.67, 1.22) | 165 | 1.06 (0.77, 1.47) |
|  | Unknown | 107 | 82 |  | 55 |  |

^1^Hazard ratios for risk of subsequent cancer, adjusted for age, gender, smoking and the variable shown

^2^Non skin cancer among first degree relatives

Other variables not shown here that were tested and not significantly associated with subsequent cancer risk are listed in the text.

Table S2. Multivariable models describing risk of melanoma after NMSC

|  |  | All NMSC | BCC | SCC |
| --- | --- | --- | --- | --- |
|  |  | N=2,243 | N=1,363 | N=880 |
|  |  | HR (95% CI) | HR (95% CI) | HR (95% CI) |
| NMSC | No  Yes | 1.0  **3.46 (1.82, 6.58)** | 1.0  **3.28 (1.66, 6.51)** | 1.0  **3.62 (1.85, 7.11)** |
|  |  |  |  |  |
| Age | Years | **1.02 (1.01, 1.05)** | **1.02 (1.00, 1.05)**^1^ | 1.02 (0.99, 1.05) |
| Sex | Male | 1.0 | 1.0 | 1.0 |
|  | Female | **0.33 (0.20, 0.56)** | **0.32 (0.17, 0.61)** | **0.36 (0.18, 0.74)** |
| Smoking | Never | 1.0 | 1.0 | 1.0 |
|  | Former | **0.57 (0.35, 0.91)** | **0.49 (0.26, 0.91)** | 0.57 (0.30, 1.09) |
|  | Current | 0.84 (0.44, 1.61) | 0.87 (0.40, 1.93) | 0.83 (0.35, 1.97) |
| Skin reaction to chronic sun exposure | Deep tan | 1.0 | 1.0 | 1.0 |
|  | Moderate tan | **2.21 (1.03, 4.70)** | 2.17 (0.90, 5.25) | **5.85 (1.38, 24.69)** |
|  | Peel | **2.64 (1.20, 5.81)** | 2.20 (0.85, 5.67) | **5.93 (1.33, 26.48)** |
|  | No tan | 1.76 (0.60, 5.13) | 1.09 (0.22, 5.47) | 4.89 (0.88, 27.25) |
| Family history NMSC | No | 1.0 | 1.0 |  |
|  | Yes | **1.60 (1.01, 2.52)** | 1.76 (0.99, 3.13) | - |

^1^Numbers are rounded; HR was statistically significant.
